# Squid meal and shrimp hydrolysate as novel protein sources for dog food

**DOI:** 10.3389/fvets.2024.1360939

**Published:** 2024-02-21

**Authors:** Joana Guilherme-Fernandes, Tiago Aires, António J. M. Fonseca, Timur Yergaliyev, Amélia Camarinha-Silva, Sofia A. C. Lima, Margarida R. G. Maia, Ana R. J. Cabrita

**Affiliations:** ^1^REQUIMTE, LAQV, ICBAS, School of Medicine and Biomedical Sciences, University of Porto, Porto, Portugal; ^2^SORGAL, Sociedade de Óleos e Rações S.A., Lugar da Pardala, S. João de Ovar, Portugal; ^3^HoLMiR – Hohenheim Center for Livestock Microbiome Research, University of Hohenheim, Stuttgart, Germany; ^4^Institute of Animal Science, University of Hohenheim, Stuttgart, Germany

**Keywords:** antioxidant activity, fecal microbiota, novel protein sources, nutritive value, palatability, pet food

## Abstract

The world’s growing pet population is raising sustainability and environmental concerns for the petfood industry. Protein-rich marine by-products might contribute to mitigating negative environmental effects, decreasing waste, and improving economic efficiency. The present study evaluated two marine by-products, squid meal and shrimp hydrolysate, as novel protein sources for dog feeding. Along with the analysis of chemical composition and antioxidant activity, palatability was evaluated by comparing a commercial diet (basal diet) and diets with the inclusion of 150 g kg^−1^ of squid meal or shrimp hydrolysate using 12 Beagle dogs (2.2 ± 0.03 years). Two *in vivo* digestibility trials were conducted with six dogs, three experimental periods (10 days each) and three dietary inclusion levels (50, 100 and 150 g kg^−1^) of squid meal or shrimp hydrolysate in place of the basal diet to evaluate effects of inclusion level on apparent total tract digestibility (ATTD), metabolizable energy content, fecal characteristics, metabolites, and microbiota. Both protein sources presented higher protein and methionine contents than ingredients traditionally used in dog food formulation. Shrimp hydrolysate showed higher antioxidant activity than squid meal. First approach and taste were not affected by the inclusion of protein sources, but animals showed a preference for the basal diet. Effects on nutrient intake reflected the chemical composition of diets, and fecal output and characteristics were not affected by the increasing inclusion levels of both protein sources. The higher ATTD of dry matter, most nutrients and energy of diets with the inclusion of both by-products when compared to the basal diet, suggests their potential to be included in highly digestible diets for dogs. Although not affected by the inclusion level of protein sources, when compared to the basal diet, the inclusion of squid meal decreased butyrate concentration and shrimp hydrolysate increased all volatile fatty acids, except butyrate. Fecal microbiota was not affected by squid meal inclusion, whereas inclusion levels of shrimp hydrolysate significantly affected abundances of Oscillosperaceae (UCG-005), Firmicutes and *Lactobacillus*. Overall, results suggest that squid meal and shrimp hydrolysate constitute novel and promising protein sources for dog food, but further research is needed to fully evaluate their functional value.

## 1 Introduction

The pet population is increasing worldwide, and, according to recent statistics, 45% of United States households own at least one dog (1), while in Europe the number of households owning dogs ranges from 5% in Turkey to 49% in Poland (2). As a result, the petfood industry is predicted to continue steadily developing (2), raising concerns about the sustainability and environmental impact of ingredient sources used in petfood production (3). Protein sources are the most expensive both in environmental and economic terms, thus being the macronutrient requiring greater attention with regard to sustainability (4). Moreover, as dogs are frequently seen as family members, pet owners are increasingly favoring palatable pet foods with high nutritional and functional values that ensure the welfare and health of their pets (5). Therefore, there is an undeniable need for alternative protein sources with lower environmental impact, while at the same time offering increased food palatability and nutritional and functional values, thus contributing to dogs’ nutritional and health status and to the sustainability of the petfood sector.

Pet owner’s mindset on food ingredients choice is also changing. According to a recent survey (6), only 19% of respondents would continue to buy pet foods with conventional protein sources, while 54% of respondents were interested in foods containing by-products, although 66% of them were not familiar with the term “by-products.” Increasing interest has emerged in alternative protein sources from terrestrial (7–10) and aquatic (11, 12) origin to replace conventional terrestrial ones due to their high nutritive value and lower environmental impact (13, 14). Under a circular economy perspective, the utilization of by-products from aquatic sources offers additional advantages as it contributes to the reduction of waste and food-feed competition, and to a greater economic and environmental efficiency (15, 16). This approach is particularly important as the volume of waste from aquatic production was reported to range from 1 to 20% for fish, from 40 to 85% for crustaceans, and from 60 to 80% for mollusks (17). The use of by-products from aquatic resources, namely crustaceans and mollusks, with high protein content and bioactive compounds (16, 18), such as carotenoids, glycosaminoglycans, bioactive peptides, and chitin/chitosan (18), which may have antioxidant, anticoagulant, antibacterial, anticancer, anti-inflammatory, and antimicrobial effects (18, 19), could be appealing to dog owners who are looking for functional pet foods (20). Furthermore, as novel protein sources, squid meal and particularly shrimp hydrolysate may play a role in the diagnosis of adverse food reactions and in the prevention of allergic reactions due to food hypersensitivity (21).

Although squid meal and shrimp hydrolysate have been studied as alternative feeds in livestock and aquaculture species, to the best of authors’ knowledge, there are no studies on the use of these by-products in dog feeding. Therefore, the aim of the current study was to evaluate the chemical composition and antioxidant activity of squid meal and shrimp hydrolysate and the effects of increasing levels of their dietary inclusion on palatability, digestibility, fecal characteristics, metabolites, and microbiota of healthy adult Beagle dogs, contributing to the recent trend in the petfood industry to provide more sustainable high protein diets with novel protein sources.

## 2 Materials and methods

Trials were approved by the Animal Ethics Committee of School of Medicine and Biomedical Sciences, University of Porto (Permit N° 344). Procedures and animal care were carried out by scientists trained by FELASA, category C, and in line with the recommendations on the ethical use of animals for scientific purposes (European Union Directive 2010/63/EU). Animals were clinically examined to ensure their suitability to participate in the studies. All dogs received regular vaccinations and were treated for endoparasites.

### 2.1 Animals and housing

Twelve Beagle dogs, six males and six females (2.2 ± 0.03 years-old; 12.6 ± 1.55 kg initial body weight, BW) were used in the palatability and digestibility assays. Sample size followed the minimum number of animals recommended for digestibility trials (22). Animals were housed in pairs in environmentally enriched communicating boxes with sliding doors to allow their individual feeding, and with inside and outside areas of 1.8 and 3.5 m^2^, respectively. Animals were allowed to daily exercise and socialize between meals in an outdoor park and had at least 30 min leash walks. During the feces collection period of the digestibility assays, animals were housed individually, had daily access to the outdoor park between meals under supervision and leash walked for at least 30 min. Kennel temperature and relative humidity were monitored daily.

### 2.2 Protein sources and experimental diets

Squid meal was provided by Inproquisa (Madrid, Spain) and comprises a by-product from the canning industry of *Dosidicus gigas* obtained through steaming and pressing for oil extraction. Shrimp hydrolysate, provided by Symrise Aqua Feed (Elven, France), resulted from the enzymatic hydrolysis of heads and cephalothoraxes of *Litopenaeus vannamei*. Both marine by-products were provided as a dry powder and kept at room temperature until use.

A commercial extruded complete diet formulated for medium size adult dogs containing (label information), animal meals, vegetable by-products, oils and fats, and beet pulp without the inclusion of squid meal and shrimp hydrolysate was used as the basal diet (SilverDog, Sorgal Pet Food, Ovar., Portugal). The experimental diets included 50, 100 or 150 g kg^−1^ of squid meal in experiment 1 (SM5, SM10, and SM15) and shrimp hydrolysate in experiment 2 (SH5, SH10, and SH15) in place of the basal diet. The studied protein sources were thoroughly mixed with the basal diet shortly before being offered to each dog. During the digestibility trials all dogs consumed the total daily food offered.

### 2.3 Palatability assays

Two-bowl tests (23) were conducted to evaluate the palatability by the pairwise comparison of the basal diet with either the experimental diet SM15 or SH15. In two consecutive days and after an overnight fast, animals (*n* = 12) were offered the choice between the two diets in two bowls placed in opposite positions (left and right, 45 cm apart) each containing half amount of the daily food allowance calculated to supply the metabolizable energy (ME) requirements of dogs (22). The bowls were placed in alternated positions between days to control side bias. The first bowl approached, and the first food tasted in each trial were recorded. Trials ended after 30 min or when the animals had consumed all the food available in a bowl. The food offered and the food refusals were weighed to calculate the ratio of consumption of the two diets.

### 2.4 Digestibility assays

The method of total fecal collection was used to assess the apparent total tract digestibility (ATTD) of the basal and experimental diets. The *in vivo* ATTD of the basal diet was determined previously to experiments 1 and 2, using 12 animals for 10 days (5 days for adaptation and 5 days for feces collection), as recommended by FEDIAF and earlier described (11). The two digestibility trials performed to evaluate the effects of inclusion levels of squid meal and shrimp hydrolysate were designed according to a replicated Latin square 3 × 3, with six animals (three males and three females, from the 12 animals used for the determination of the *in vivo* digestibility of the basal diet), three experimental periods of 10 days (5 days for adaptation to the diet and 5 days for total feces collection) and three dietary inclusion levels (50, 100 or 150 g kg^−1^).

At the beginning of each adaptation period and prior to the morning feeding, the animals were weighed and the body condition was assessed according to a 9 point-scale, with 5 considered the ideal body condition score (24). Daily food allowance was defined according to the ME requirements considering the ideal BW of individuals, ME (kcal/day) = 110 × BW^0.75^ (22), and adjusted to body condition score. Animals were individually fed their daily ration in two equal meals (8.30 a.m. and 5.00 p.m.). Fresh water was provided *ad libitum*. During the feces collection periods, the number of defecations were recorded and individual fresh feces were weighed and scored with a 5-point scale to evaluate the consistency of stools, with score (1) reflecting watery diarrhea, (3.5) firm, shaped, and dry stools, and (5) powdery hard mass pellets (25). Diarrhea was scored from 1 to 2, according to the scale. Fecal samples were mixed, subsampled, and stored in plastic bags at −20°C until analysis of chemical composition, pH, ammonia-N and volatile fatty acids (VFA) concentrations and fecal microbiota. Analyses were carried out in fecal samples pooled per dog and period.

### 2.5 Analytical procedures

#### 2.5.1 Proximate analysis

Protein sources, basal diet and fecal samples were dried until constant weight in an air-forced oven at 65°C, 1-mm milled, and analyzed in duplicate, according to official methods (26), as previously described (27). Samples were analyzed for dry matter (DM; ID 934.01), ash (ID 942.05), ether extract (EE; ID 920.39), and Kjeldahl N (ID 990.03; in fresh feces samples). Crude protein (CP) was calculated as Kjeldahl N × 6.25. Gross energy (GE) analysis was performed with an adiabatic bomb calorimeter (Werke C2000, IKA, Staufen, Germany). The basal diet was also analyzed for neutral detergent fiber (with α-amylase, without sodium sulfite, and expressed exclusive of residual ash, NDF) (28), and for starch (in 0.5-mm milled samples) (29) contents.

Amino acids were determined as described by Aragão et al. (30). Briefly, samples were hydrolyzed with 6 M HCl solution at 116°C for 48 h. Precolumn derivatization was performed according to the AccQ Tag method (Waters, Milford, MA, United States) using the Waters AccQ Fluor Reagent (6-aminoquinolyl-N-hydroxysuccinimidyl carbamate) and the analyses carried out by ultra-high-performance liquid chromatography on a Waters reversed-phase amino acid analysis system with norvaline as the internal standard. Peaks were then analyzed with EMPOWER software (Waters). The analyses were carried out in duplicate.

#### 2.5.2 Antioxidant activity assays

Squid meal and shrimp hydrolysate extracts were prepared, in quadruplicate, as reported by Zaharah and Rabeta (31). A volume of 20 mL of Milli-Q water was added to 2 g of squid meal and to 0.8 g of shrimp hydrolysate. Samples were then incubated in an orbital shaker overnight, in the dark, at 160 rpm and 27°C, and centrifuged for 30 min at 2,500 rpm at 20°C. The supernatant was collected and diluted to obtain a final concentration based on the initial dry weight and the final supernatant volume yielding 2.5 mg mL^−1^ for squid meal extracts and 1 mg mL^−1^ for shrimp hydrolysate. The antioxidant activity was determined through the 2,2-azinobis-(3-ethylbenzothiazoline-6-sulfonic acid) radical cation decolorization test (ABTS assay) (32), the scavenging activity of 2,2-diphenyl-1-picrylhydrazyl radical (DPPH assay) (33), the ferric reducing antioxidant power (FRAP assay), and the Folin–Ciocalteu reducing capacity (FC assay) (34). For the ABTS assay, ABTS solution was prepared with equal volumes of 7 mM ABTS (Sigma-Aldrich, A1888, Saint Louis, MO, United States) and 2.45 mM potassium persulfate solution and left overnight in dark at room temperature. ABTS solution was diluted in water to achieve an absorbance of 0.90 ± 0.02 at 734 nm (35). In a 96 well plate, 50 μL of ABTS was added to 50 μL of each sample and solvent (blank), and absorbance was assessed after an incubation period of 30 min at 25°C in the dark. For the DPPH assay, a 0.2 mM DPPH solution (Sigma-Aldrich, D9132) was freshly prepared with methanol. In a 96 well plate, 100 μL of DPPH solution was added to 100 μL of each sample and solvent (blank). Absorbance was read at 517 nm after an incubation period of 30 min in the dark at 22°C. For the FRAP assay, a fresh FRAP solution was prepared by mixing 10 mM TPTZ stock solution, acetate buffer (300 mM at pH of 3.6), and 20 mM FeCl_3_ solution in a proportion of 10:1:1 (v/v/v). Samples were incubated for 30 min at 37°C in the dark in a 96 well plate, preceding the addition of 300 μL of FRAP solution to 10 μL of samples diluted in 30 μL of Milli-Q water. The absorbance was read at 593 nm. Finally, for the FC assay, in a 96 well plate, 12 μL of Folin–Ciocalteu phenol reagent (Sigma-Aldrich, F9252) was added to 15 μL of samples diluted in 170 μL of Milli-Q water, followed by the addition of 30 μL of Na_2_CO_3_ solution (10% w/v). After a period of 1 h of incubation in the dark at room temperature, 73 μL of Milli-Q water was added to each well and absorbance was read at 765 nm. Absorbance of samples, solvent (blank), and standard solution (quercetin from Sigma-Aldrich, Q4951) were measured using a Synergy™ HT Multimode plate reader (BioTek^®^ Instruments Inc., Winooski, VT, United States). Analyses were performed in triplicate. A calibration curve with the standard solution was performed in all assays and the results were expressed in milligram of quercetin equivalents per gram of DM (mg Q g^−1^ DM).

#### 2.5.3 Fecal end-fermentation products

The fecal pH was measured with a potentiometer (pH and Ion-Meter GLP 22, Crison, Barcelona, Spain) after dilution of thawed feces to 1:10 (w/v) in water and incubation for 10 min in a sonication bath at room temperature. The concentration of fecal ammonia-N was determined according to the methodology of Chaney and Marbach (36) adapted to dog feces. Briefly, fecal samples were thawed, diluted to 1:10 (w/v) in 2 M KCl and centrifuged at 5200 g at 4°C for 60 min. The supernatant was filtered with a 0.45 μm pore size polyethersulfone syringe filter (FILTER-LAB, Barcelona, Spain). Forty μL of water were added to 40 μL of sample, followed by the addition of 2.5 mL of phenol solution and 2 mL of 0.37% alkaline hypochlorite solution. Samples were firstly incubated for 10 min at 37°C, followed by 40 min at 22°C in the dark. An ammonia solution (32 mg dL^−1^) was used as standard. The absorbance was read at 550 nm in a Synergy™ HT Multimode plate reader (BioTek^®^ Instruments Inc.). Analysis was done in duplicate.

For VFA analysis, fecal samples were diluted to 1:10 (w/v) in 25% ortho-phosphoric acid solution with an internal standard (4 mM 3-methyl valerate, Sigma-Aldrich), and centrifuged for 60 min at 2360 g at 4°C. The supernatant was filtered with a 0.45 μm pore size polyethersulfone syringe filter (FILTER-LAB) and analyzed by gas chromatography as described by Pereira et al. (37).

#### 2.5.4 Fecal microbiota

DNA from fecal samples was extracted by Fast DNA™ Spin Kit for soil and quantified using a NanoDrop 2000 spectophotometer (Thermo Scientific, Waltham, MA, United States). For bacterial amplicons library preparation, V1–V2 hypervariable regions of the 16S rRNA gene were amplified (38). Unique barcodes (6-nt) were linked to forward primers, while index adapters were attached to reverse. 16S library was created by two rounds of polymerase chain reaction (PCR). In short, 1 μL of extracted DNA was used for the first round of PCR, in a total of 20 μL reaction mix volume, with 0.2 μL of PrimeSTAR HS DNA polymerase and 0.5 μL of each forward and reverse primers (in the concentration of 0.2 μM each). The second round of PCR was performed with 1 μL of the first PCR product with a total volume of 50 μL. An initial denaturation was performed at 95°C for 3 min and was followed by 15 cycles for the first round of PCR and 20 cycles for the second, with denaturation at 98°C (10 s) and subsequent annealing at 55°C (10 s), elongation at 72°C (45 s) and a final extension at 72°C (2 min). Library normalization was carried out by the SequalPrepTM Normalization Kit (Invitrogen Inc., Carlsbad, CA, United States) and sequencing was performed with the 250 bp paired-end Illumina NovaSeq 6000 platform.

Raw sequences were demultiplexed with Sabre.^1^ Downstream analyses were implemented using Qiime2 (39). Primers/adapters trimming was performed by the q2-cutadapt plugin (40). Denoising, quality filtering, merging of paired reads, and chimeras removal were completed by the q2-dada2 (41). Taxonomy assignation of amplicon sequence variants (ASVs) was performed with VSEARCH-based consensus (42) and pre-fitted sklearn-based classifiers (43) against the Silva database (v138.1, 16S 99%) (44). The reference reads were preprocessed by RESCRIPt (45). A phylogenetic tree was built by the q2-phylogeny, utilizing MAFFT (v7.3) (46) and FastTree (v2.1) (47). Alpha diversity was assessed by Shannon’s entropy (48) and Faith’s phylogenetic diversity (49) indices, and beta diversity by Jaccard (50) and Bray-Curtis (51) distances. Beta diversity ordination was performed by principal-coordinate analysis (PCoA) (52). Alpha diversity metrics were compared by the Wilcoxon test (53), and beta diversity distances by the Adonis test (999 permutations) (54). Differentially abundant genera (only for counts of genera with relative abundance ≥1% and prevalence ≥10%) were detected by ALDEx2 (55). All *p*-values obtained from multiple comparisons were adjusted using the Benjamini-Hochberg procedure (56). Raw sequences are available at the European Nucleotide Archive (ENA) under accession number PRJEB71521.

### 2.6 Calculations and statistical analysis

A Chi-square test was used to analyze the first approach and first taste results from the two-bowl tests, being the ratio of consumption analyzed through a paired *t*-test, both at 5% probability level (*n* = 12).

Fecal production was calculated as:

$$\mathrm{Fecal}\;\mathrm{production}\;\left(\%\right)=\frac{\mathrm{dried}\;\mathrm{fecal}\;\mathrm{output}\;\left(\frac{g}{d}\right)}{\mathrm{dry}\;\mathrm{matter}\;\mathrm{intake}\;\left(\frac{g}{d}\right)}\;x\;100$$

The ATTD of the basal and experimental diets was determined using the following equation:

$$\mathrm{ATTD}\;\left(\%\right)=\frac{\mathrm{nutrient}\;\mathrm{intake}\;\left(\frac{g}{d}\right)-\mathrm{fecal}\;\mathrm{nutrient}\;\left(\frac{g}{d}\right)}{\mathrm{nutrient}\;\mathrm{intake}\;\left(\frac{g}{d}\right)}\;x\;100$$

Metabolizable energy content of diets was calculated according to FEDIAF (22), as follows:

$$\begin{array}{l} \mathrm{ME}\;\left(MJ/\mathrm{kg}\;DM\right)\\ =\frac{\left(GE\;\mathrm{intake}\;\left(\frac{MJ}{d}\right)\;-\mathrm{fecal}\;GE\;\left(\frac{MJ}{d}\right)\right)-\left(CP\;\mathrm{intake}\;\left(\frac{g}{d}\right)-\mathrm{fecal}\;CP\;\left(\frac{g}{d}\right)\right)\;x\;5.23}{DM\;\mathrm{intake}\;\left(\frac{g}{d}\;\right)} \end{array}$$

For each digestibility trial, data on BW, body condition score, diet and nutrient intake, ATTD, ME content, fecal output and characteristics, and fecal metabolites were analyzed according to a replicated 3 × 3 Latin square considering the fixed effects of square, dog within the square, period, inclusion level of protein source and the residual error (SAS, 2022, release 3.81., SAS Institute Inc., Cary, NC, United States). Means were compared by the least significant difference test when significant differences (*p* < 0.05) among experimental diets were found. A paired *t*-test was performed to compare the basal diet with experimental diets with inclusion of squid meal or shrimp hydrolysate (SAS, 2022, release 3.81.) to mimic the at-home scenario of dog owners changing the diet of their animals, thus understand the perceived effects. For that, data from the six dogs collected during the digestibility trial with each studied protein source were used for comparison with the values obtained for the same animals during the digestibility trial on the basal diet.

## 3 Results

### 3.1 Chemical composition

The proximate composition of protein sources, basal diet, and experimental diets with increasing levels of squid meal and shrimp hydrolysate is shown in Table 1. Compared to shrimp hydrolysate, squid meal presented a higher content of CP (658 g kg^−1^ DM and 810 g kg^−1^ DM, respectively), and lower ash (151 g kg^−1^ DM and 103 g kg^−1^ DM) and EE (93.8 g kg^−1^ DM and 31.2 g kg^−1^ DM) contents. The basal diet presented 252 g kg^−1^ CP (DM basis) and 91.4 g kg^−1^ EE (DM basis). The chemical composition of the experimental diets of both experiments reflected the chemical composition of the basal diet and the studied protein sources.

**Table 1:** Proximate composition (g kg^−1^ dry matter, DM) and gross energy (MJ kg^−1^ DM) of protein sources, basal diet, and experimental diets with increasing levels of inclusion of squid meal (Experiment 1) and shrimp hydrolysate (Experiment 2) in substitution of the basal diet.

	Protein sources		Basal diet	Experiment 1			Experiment 2		
Item	Squid meal	Shrimp hydrolysate		SM5	SM10	SM15	SH5	SH10	SH15
DM, g kg^−1^	937	962	924	925	925	926	926	928	930
Ash	103	151	125	124	123	122	126	128	129
Crude protein	810	658	252	280	308	336	272	293	313
Ether extract	31.2	93.8	91.4	88.4	85.4	82.4	91.5	91.6	91.8
Neutral detergent fiber	ND	ND	228	217	205	194	217	205	194
Starch	ND	ND	311	295	280	264	295	280	264
Gross energy	21.0	21.3	183	175	167	159	175	167	159

ND, not determined.

Squid meal and shrimp hydrolysate presented 652 g kg^−1^ DM and 445 g kg^−1^ DM total amino acids content, being the main essential and non-essential amino acids found in both studied protein sources, respectively, arginine, lysine and leucine, and glutamic acid plus glutamine and tyrosine (Table 2).

**Table 2:** Total, essential and non-essential amino acids (g kg^−1^ DM) of protein sources, basal diet, and experimental diets with increasing levels of inclusion of squid meal (Experiment 1) and shrimp hydrolysate (Experiment 2) in substitution of the basal diet.

Item	Protein sources		Basal diet	Experiment 1			Experiment 2		
	Squid meal	Shrimp hydrolysate		SM5	SM10	SM15	SH5	SH10	SH15
*Essential amino acids*									
Arginine	57.9	31.6	20.50	22.4	24.2	26.1	21.1	21.6	22.2
Histidine	14.0	8.8	6.43	6.81	7.19	7.56	6.55	6.67	6.79
Lysine	54.4	38.5	16.00	17.9	19.8	21.8	17.1	18.3	19.4
Threonine	27.9	17.5	10.50	11.4	12.2	13.1	10.9	11.2	11.6
Isoleucine	26.0	17.6	9.63	10.4	11.3	12.1	10.0	10.4	10.8
Leucine	45.7	30.3	21.20	22.4	23.7	24.9	21.7	22.1	22.6
Valine	33.1	30.0	16.20	17.0	17.9	18.7	16.9	17.6	18.3
Methionine	29.7	16.3	3.68	4.98	6.28	7.58	4.31	4.95	5.58
Methionine + cystine	34.1	18.9	8.24	9.53	10.8	12.1	8.77	9.30	9.84
Phenylalanine	27.1	24.0	12.2	12.9	13.7	14.4	12.8	13.4	14.0
Phenylalanine + tyrosine	84.1	53.9	19.0	22.3	25.5	28.8	20.8	22.5	24.2
Total	316	215	116	126	136	146	121	126	131
*Non-essential amino acids*									
Cystine	4.41	2.54	4.56	4.55	4.55	4.54	4.46	4.36	4.26
Tyrosine	57.0	29.9	6.82	9.33	11.8	14.4	7.97	9.13	10.3
Aspartic acid + Asparagine	42.4	29.3	21.8	22.8	23.9	24.9	22.2	22.6	22.9
Glutamic acid + Glutamine	75.8	53.4	39.9	41.7	43.5	45.3	40.6	41.2	41.9
Alanine	34.9	34.2	19.4	20.2	21.0	21.7	20.1	20.9	21.6
Glycine	41.2	30.5	26.7	27.4	28.2	28.9	26.9	27.1	27.3
Proline	51.6	34.3	24.8	26.1	27.5	28.8	25.3	25.7	26.2
Serine	28.6	16.7	15.5	16.2	16.8	17.5	15.6	15.6	15.7
Total	336	231	159	168	177	186	163	167	170

### 3.2 Antioxidant activity

The antioxidant activity of shrimp hydrolysate was higher than that of squid meal in all the assays performed (19.8 vs. 4.35 mg Q g^−1^ DM for ABTS, 10.4 vs. 1.58 mg Q g^−1^ DM for FC, and 2.27 vs. 0.36 mg Q g^−1^ DM for FRAP; Table 3). No reaction in the DPPH assay was observed with squid meal extract.

**Table 3:** Antioxidant activity of protein sources extracts expressed in milligram of quercetin, Q, per gram of dry matter.

Protein sources extracts	Quercetin equivalent (mg Q g^−1^ DM)			
	ABTS assay	DPPH assay	FC assay	FRAP assay
Squid meal	4.35 ± 0.23	ND	1.58 ± 0.09	0.36 ± 0.03
Shrimp hydrolysate	19.8 ± 0.03	1.90 ± 0.120	10.4 ± 0.23	2.27 ± 0.084

Data expressed as mean ± SD; ND, reaction not detected.

### 3.3 Palatability assays

**Figure 1:**
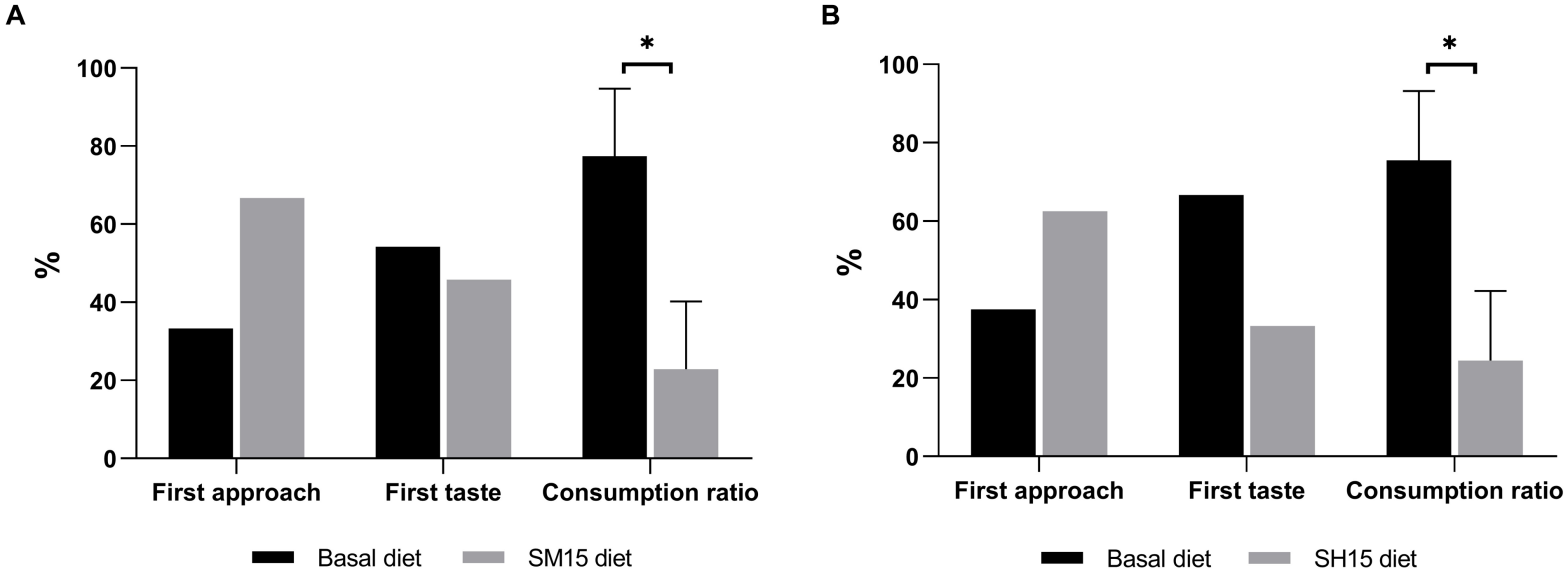
Frequency of first approach and first taste (mean, *n* = 12), and consumption ratio (mean ± SEM, *n* = 12) of basal diet in comparison with either SM15 diet **(A)** or SH15 diet **(B)** in the two-bowl tests. ^*^p < 0.05.

The results of the two-bowl tests are shown in Figure 1. No differences were found on first diet approached and tasted in both tests. The consumption of either SM15 (22.9%) or SH15 (24.5%) was significantly lower (*p* < 0.05) in comparison with the consumption of the basal diet (77.4 and 75.5%, respectively for squid meal and shrimp hydrolysate tests).

### 3.4 Digestibility assays

Dogs remained healthy throughout the studies with no episodes of emesis, diarrhea, and food refusal.

#### 3.4.1 Experiment 1: squid meal

Increasing levels of inclusion of squid meal kept unaffected diet intake, and significantly increased (*p* < 0.05) intake of organic matter (OM), CP and GE while decreasing EE intake (Table 4). No effects of squid meal inclusion levels were observed on fecal output and characteristics, and ATTD of DM, nutrients and energy and ME content, except for ATTD of CP that was significantly higher (*p* = 0.024) in SM15 diet (79.1%). Fecal metabolites were not affected by the level of squid meal inclusion, with the only exception of fecal pH, that was the highest with SM15 and the lowest with SM10 (*p* = 0.024).

**Table 4:** Experiment 1: body weight, body condition score, diet, nutrient and energy intake, apparent total tract digestibility (ATTD), metabolizable energy content, fecal output and characteristics, and fecal metabolites of dogs fed experimental diets with squid meal inclusion and basal diet (*n* = 6).

Item	Experimental diets^1^			SEM	*p*-value	Basal diet	Basal diet vs. Experimental diets^2^
	SM5	SM10	SM15				*p*-value
Body weight	13.1	12.7	12.6	0.27	0.479	12.5 ± 1.73	0.167
Body condition score	5.50	5.33	5.33	0.192	0.785	5.33 ± 0.471	0.717
*Diet intake*							
g diet d^−1^, as-is	316.6	316.6	316.5	0.13	0.730	316.7 ± 42.13	0.071
g diet d^−1^, dry matter (DM)	292.7	292.9	293.0	0.13	0.199	292.6 ± 38.92	0.020
*Nutrient intake, g d^−1^*							
Organic matter	256.3^a^	256.9^b^	257.3^c^	0.12	0.002	255.9 ± 34.04	<0.001
Crude protein	82.0^a^	90.4^b^	98.7^c^	0.50	<0.001	73.7 ± 9.81	<0.001
Ether extract	25.9^c^	25.0^b^	24.1^a^	0.05	<0.001	26.7 ± 3.56	<0.001
Gross energy, MJ d^−1^	5.40^a^	5.45^b^	5.49^c^	0.004	<0.001	5.36 ± 0.007	<0.001
*Fecal output and characteristics*							
g feces d^−1^, as-is	285	279	270	6.0	0.268	300 ± 56.4	0.006
g feces d^−1^, DM	85.1	86.0	81.8	2.12	0.378	93.0 ± 13.67	0.001
Gross energy, MJ d^−1^	1.18	1.24	1.14	0.046	0.346	1.36 ± 0.214	<0.001
Production, %	28.9	29.4	27.9	0.73	0.388	31.8 ± 2.33	0.001
DM, %	30.4	30.9	30.7	0.26	0.401	31.8 ± 1.72	0.002
Consistency score	3.29	3.31	3.09	0.061	0.065	3.35 ± 0.297	0.021
Defecations no. d^−1^	2.60	2.43	2.50	0.098	0.508	2.63 ± 0.243	0.207
*ATTD, %*							
DM	71.1	71.0	73.3	0.72	0.099	68.2 ± 2.33	<0.001
Organic matter	76.9	76.6	78.4	0.64	0.173	74.4 ± 2.13	<0.001
Crude protein	74.8^a^	75.7^a^	79.1^b^	0.91	0.024	69.2 ± 3.90	<0.001
Ether extract	90.6	90.2	90.9	0.39	0.463	91.1 ± 0.99	0.067
Gross energy	78.3	77.2	79.3	0.80	0.245	74.6 ± 2.38	<0.001
Metabolizable energy, MJ kg^−1^	13.3	13.1	13.5	0.14	0.317	12.8 ± 0.39	<0.001
*Fecal metabolites, mg kg^−1^, DM*							
pH	6.87^a,b^	6.82^a^	6.94^b^	0.025	0.024	6.96 ± 0.122	0.006
Ammonia-N	175	183	170	10.2	0.690	138 ± 13.5	<0.001
*VFA*							
Total	785	729	703	0.1	0.443	766 ± 81.0	0.659
Acetate	471	437	427	0.1	0.486	447 ± 54.9	0.965
Propionate	211	202	188	0.1	0.532	209 ± 23.5	0.629
Butyrate	68.5	59.9	57.3	0.01	0.157	78.3 ± 16.11	0.011
*Iso*-butyrate	11.7	10.3	9.21	0.001	0.218	10.6 ± 2.11	0.867
*Iso*-valerate	14.8	13.3	12.4	0.01	0.405	11.3 ± 1.93	0.111
Valerate	2.42	2.76	2.86	0.002	0.539	3.38 ± 1.513	0.121
*Iso*-caproate	5.01	3.27	5.32	0.001	0.375	5.46 ± 2.220	0.222
Caproate	1.34	1.09	1.21	0.000	0.891	1.28 ± 0.352	0.736
Acetate:propionate	2.25	2.21	2.32	0.060	0.495	2.14 ± 0.103	0.016

^1^SM5, diet with 5% inclusion of squid meal; SM10, diet with 10% inclusion of squid meal; SM15, diet with 15% inclusion of squid meal. ^2^Data from the same dogs used in the squid meal digestibility trial. ^a,b,c^Values with different superscript letters in the same row are significantly different (*p* < 0.05).

Compared to the basal diet, the dietary inclusion of squid meal significantly increased (*p* < 0.05) DM intake and ATTD of DM, OM, CP, and GE as well as ME content, but decreased EE intake and fecal output. Fecal DM and GE content, consistency score, pH and butyrate content were significantly lower (*p* < 0.05) whereas ammonia-N concentration and acetate:propionate ratio were higher in diets with squid meal inclusion compared to the basal diet (Table 4).

#### 3.4.2 Experiment 2: shrimp hydrolysate

Increasing levels of inclusion of shrimp hydrolysate significantly increased (*p* < 0.05) the intake of DM, OM, CP, EE, and GE, but decreased fecal GE, with no differences being observed on ATTD of DM, nutrients, and energy, ME content, and fecal output, characteristics, and metabolites (Table 5). Compared to the basal diet, the inclusion of shrimp hydrolysate significantly increased (*p* < 0.05) intake of DM, CP, EE, and GE, ATTD of DM, OM, CP, and GE, ME content, total VFA production and the concentration of the individual VFA (except butyrate) and decreased fecal output, consistency score and the number of defecations (Table 5).

**Table 5:** Experiment 2: body weight, body condition score, diet, nutrient and energy intake, apparent total tract digestibility (ATTD), metabolizable energy content, fecal output and characteristics, and fecal metabolites of dogs fed experimental diets with shrimp hydrolysate inclusion and basal diet (*n* = 6).

Item	Experimental diets^1^			SEM	*p*-value	Basal diet	Basal diet versus Experimental diets^2^
	SH5	SH10	SH15				*p*-value
Body weight	12.9	13.1	13.0	0.21	0.768	12.6 ± 1.19	0.075
Body condition score	5.67	5.33	5.33	0.173	0.342	5.17 ± 0.373	0.056
*Diet intake*							
g diet d^−1^, as-is	312.2	312.1	312.6	0.13	0.051	312.6 ± 33.41	0.148
g diet d^−1^, dry matter (DM)	289.0^a^	289.5^b^	290.6^c^	0.12	<0.001	288.7 ± 30.87	<0.001
*Nutrient intake, g d^−1^*							
Organic matter	252.4^a^	252.5^a^	253.0^b^	0.10	0.004	252.5 ± 27.00	0.549
Crude protein	78.9^a^	85.1^b^	91.5^c^	0.25	<0.001	72.8 ± 7.78	<0.001
Ether extract	26.4^a^	26.5^b^	26.7^c^	0.01	<0.001	26.4 ± 2.82	<0.001
Gross energy, MJ d^−1^	5.34^a^	5.40^b^	5.46^c^	0.003	<0.001	5.29 ± 0.006	<0.001
*Fecal output and characteristics*							
g feces d^−1^, as-is	266	262	253	3.8	0.117	282 ± 25.3	<0.001
g feces d^−1^, DM	85.9	82.7	82.9	1.74	0.398	88.8 ± 8.17	0.002
Gross energy, MJ d^−1^	1.22^b^	1.18^a^	1.15^a^	0.011	0.007	1.27 ± 0.116	<0.001
Production, %	29.7	28.7	28.6	0.55	0.356	28.5 ± 0.86	0.248
DM, %	32.8	32.0	33.0	0.67	0.561	32.2 ± 1.42	0.472
Consistency score	3.44	3.20	3.25	0.061	0.052	3.54 ± 0.286	0.006
Defecations no. d^−1^	2.53	2.53	2.37	0.081	0.295	2.70 ± 0.396	0.009
*ATTD, %*							
DM	69.9	71.3	71.4	0.55	0.156	69.2 ± 0.93	0.001
Organic matter	76.5	77.4	77.5	0.38	0.182	75.3 ± 0.67	<0.001
Crude protein	75.7	76.9	77.3	0.76	0.357	71.9 ± 1.36	<0.001
Ether extract	92.5	92.3	91.8	0.26	0.178	92.1 ± 0.86	0.799
Gross energy	77.1	78.0	78.4	0.39	0.124	76.0 ± 0.65	<0.001
Metabolizable energy, MJ kg^−1^	13.2	13.3	13.5	0.07	0.064	13.0 ± 0.11	<0.001
*Fecal metabolites, mg kg^−1^, DM*							
pH	6.98	6.97	6.99	0.052	0.969	7.08 ± 0.197	0.062
Ammonia-N	151	147	142	7.7	0.745	159 ± 45.1	0.223
*VFA*							
Total	943	937	1,030	0.1	0.363	720 ± 111.5	<0.001
Acetate	547	549	602	0.1	0.368	414 ± 80.6	<0.001
Propionate	248	255	267	0.1	0.659	207 ± 30.1	<0.001
Butyrate	69.6	61.0	64.8	0.01	0.569	71.4 ± 15.18	0.115
*Iso*-butyrate	20.8	18.6	22.5	0.01	0.397	9.71 ± 1.474	<0.001
*Iso*-valerate	27.4	24.3	34.3	0.01	0.380	11.3 ± 0.97	<0.001
Valerate	11.6	8.79	12.7	0.002	0.529	3.30 ± 1.725	<0.001
*Iso*-caproate	12.8	13.6	14.6	0.01	0.802	3.32 ± 0.696	<0.001
Caproate	5.78	6.82	11.9	0.002	0.073	0.835 ± 0.2792	<0.001
Acetate:propionate	2.22	2.16	2.26	0.079	0.677	1.99 ± 0.173	<0.001

^1^SH5, diet with 5% inclusion of shrimp hydrolysate; SH10, diet with 10% inclusion of shrimp hydrolysate; SH15, diet with 15% inclusion of shrimp hydrolysate. ^2^Data from the same dogs used in the shrimp hydrolysate digestibility trial. ^a,b,c^Values with different superscript letters in the same row are significantly different (*p* < 0.05).

### 3.5 Fecal microbiota

**Figure 2:**
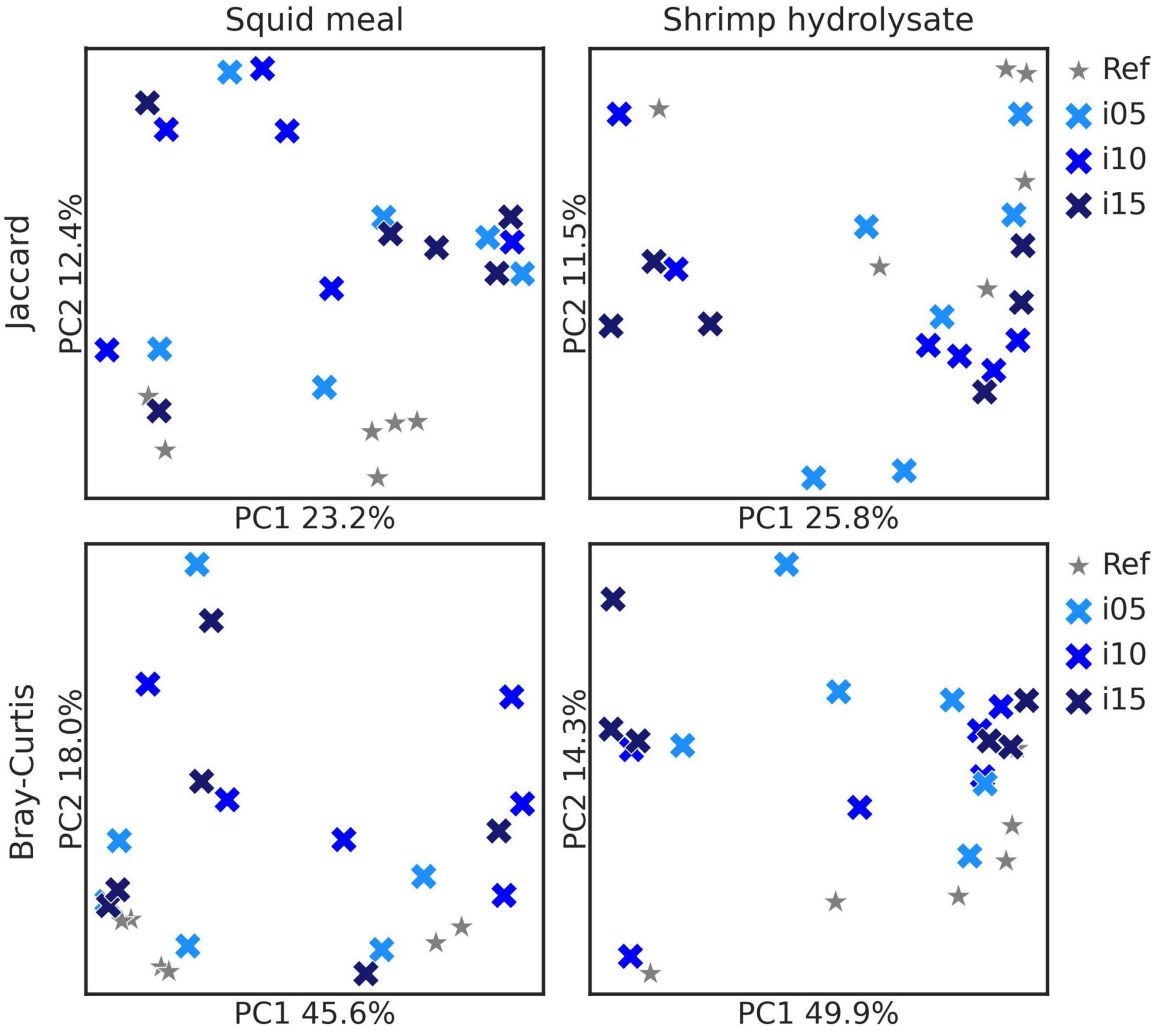
Beta diversity metrics. Principal-coordinate analysis (PCoA) based on Jaccard and Bray-Curtis distances of fecal bacteria of dogs fed the basal diet (ref), and the experimental diets with increasing levels of inclusion of squid meal or shrimp hydrolysate (i05, i10, i15) in place of the basal diet. Each cross indicates one sample. Basal diet and experimental diets are differentiated by shapes and color and inclusion levels by the color gradient.

**Figure 3:**
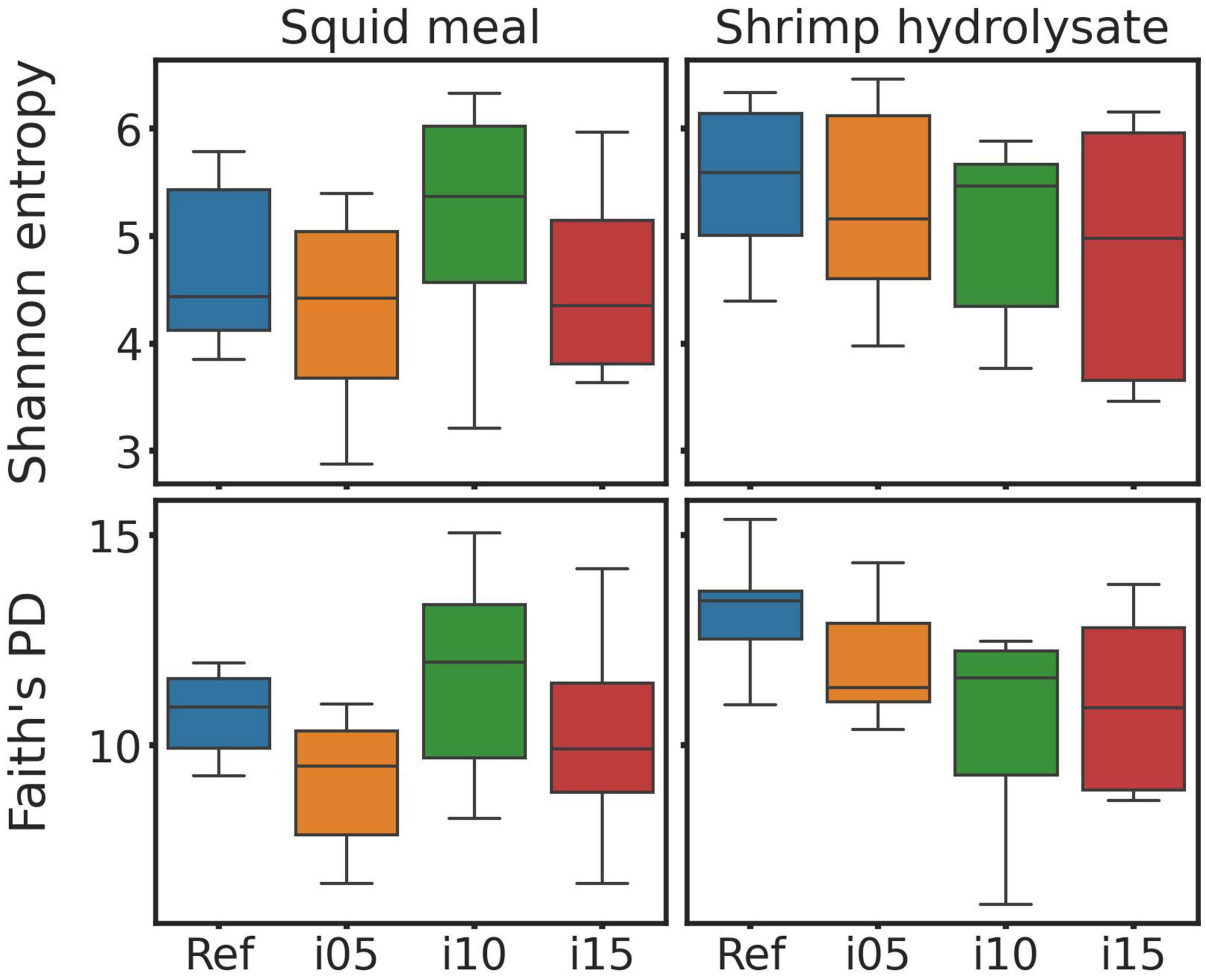
Alpha diversity metrics. Bloxpots of Sannon entropy and Faith’s PD indices of fecal bacteria of dogs fed the basal diet (ref), and the experimental diets with increasing levels of inclusion of squid meal or shrimp hydrolysate (i05, i10, i15) in place of the basal diet.

**Figure 4:**
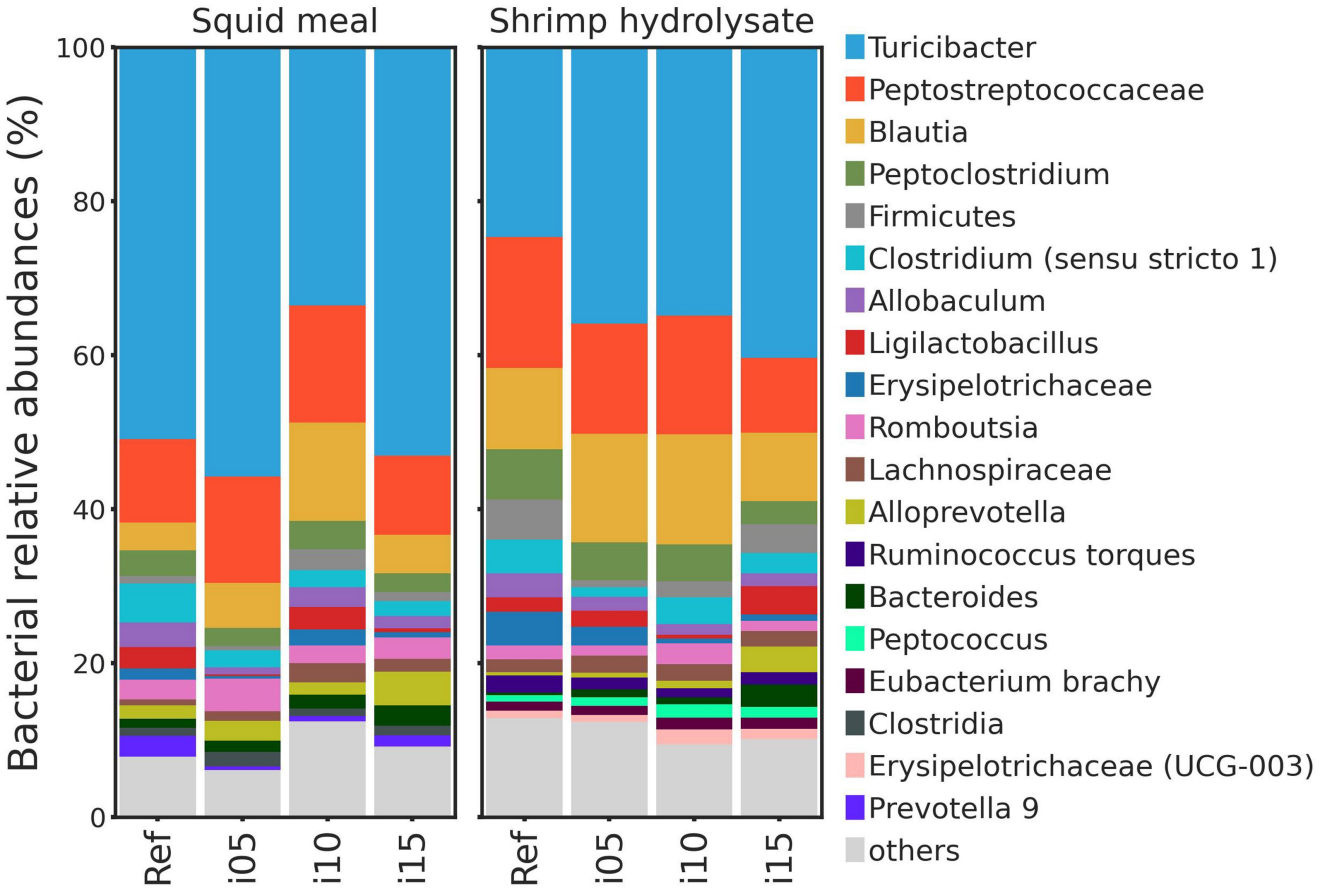
Bacterial relative abundance (%). Taxonomy barplots at the genus level of dogs fed the basal diet (ref), and the experimental diets with increasing levels of inclusion of squid meal or shrimp hydrolysate (i05, i10, i15) in place of the basal diet. If genus level was not assigned, the last available taxonomy rank was used for the label.

**Figure 5:**
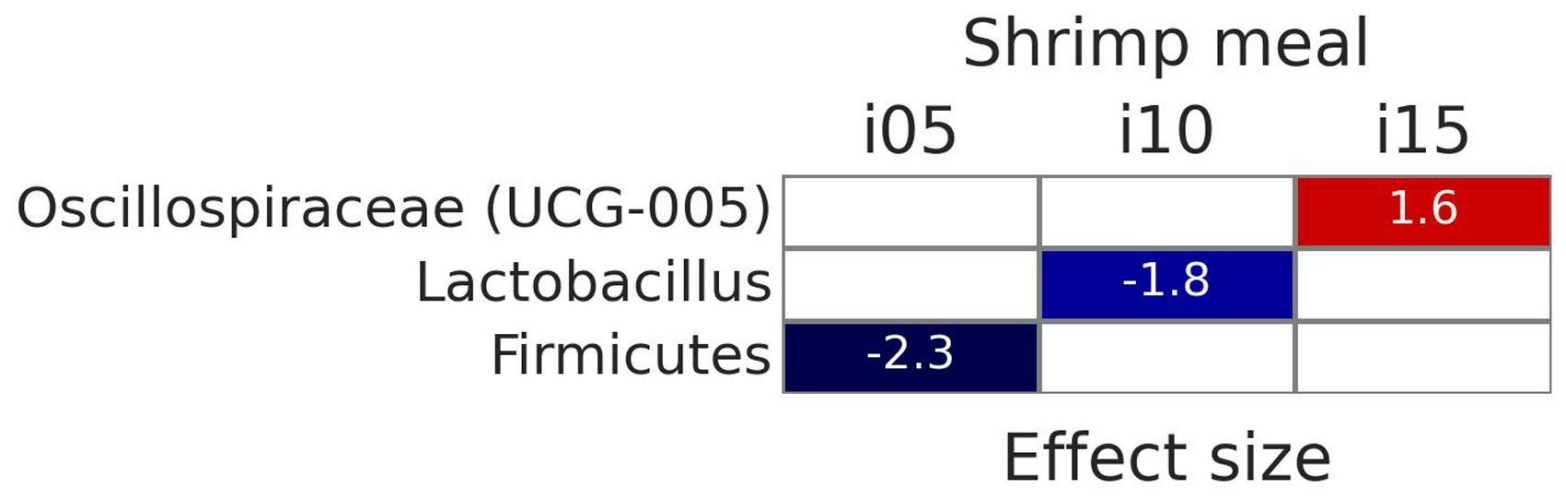
Differentially abundant genera (*p* < 0.05), according to ALDEx2, of fecal bacteria of dogs fed the experimental diets with increasing levels of inclusion of shrimp hydrolysate (i05, i10, i15) in place of the basal diet in comparison with the basal diet. The value in red indicates increased abundance and the values in blue indicate decreased abundance.

To assess samples distribution based on bacterial communities, PCoA plots were created using Jaccard and Bray-Curtis distances (Figure 2). The Adonis test revealed no significant effect of including squid meal or shrimp hydrolysate in diets for both Jaccard and Bray-Curtis metrics. Regarding alpha diversity, no effect of the inclusion of protein sources in diets on Shannon entropy and Faith’s phylogenetic diversity was detected by the Wilcoxon test (Figure 3). In the feces of dogs fed the basal and experimental diets, *Turicibacter* was the most abundant genus, followed by unclassified members of Peptostreptococcaceae and *Blautia* (Figure 4). When tested with ALDEx2, the experimental diet SH15 resulted in increased abundances of Oscillospiracea (UCG-005 in Silva database) in comparison to the basal diet, while SH5 and the SH10 experimental diets, respectively, decreased abundances of Firmicutes and *Lactobacillus* (Figure 5). According to the same test, no differentially abundant genera were discovered between dog feces fed the basal diet and experimental diets with squid meal inclusion.

## 4 Discussion

The European Union legislation allows the use of crustaceans and mollusks in petfood (57) and no constrains were found to be imposed by the Association of American Feed Control Officials (58). However, the use of protein-rich by-products obtained from these aquatic organisms in petfood has not been studied yet, so the present study aimed to evaluate the chemical composition, including the amino acid profile, and antioxidant activity of squid meal and shrimp hydrolysate, and the effect of increasing levels of their dietary inclusion on the palatability, digestibility, fecal characteristics and metabolites, and microbiota in adult healthy dogs.

### 4.1 Chemical composition

The chemical composition of the experimental diets in both experiments reflected the chemical composition of the protein studied sources, namely by enhancing the CP and amino acid content compared to the basal diet. All diets met the nutritional requirements for adult dogs (22).

Protein was the main chemical constituent of the studied by-products, with squid meal presenting the highest amount. The CP content of squid meal agrees with the wide range of values reported in the literature (680 g kg^−1^ DM (59) to 805 g kg^−1^ DM (60)), as well as for shrimp hydrolysate (436 g kg^−1^ DM (61) to 888 g kg^−1^ DM (62)). Both by-products, particularly the squid meal, presented higher CP content than animal and vegetal protein sources commonly used in dog food formulation such as poultry by-product meal (590 g kg^−1^ DM), meat and bone meal (509 g kg^−1^ DM), corn gluten meal (563 g kg^−1^ DM), and soybean meal (445 g kg^−1^ DM) (63).

The CP content of both protein sources was higher than the total amino acid content, as previously reported in related sources (64, 65), questioning the use of 6.25 as the conversion factor to calculate CP. As no recommendations exist regarding the conversion factor to be applied to these sources, amino acid analysis is considered more suitable than CP to evaluate the protein content of foods, as suggested by FAO (66). The total content of amino acid of squid meal observed in the current study corresponds to the range of values previously reported [457 g kg^−1^ DM (65) to 628 g kg^−1^ DM (67)], but the amino acid profile differs from comparable sources. While in the present study, the essential amino acids found at the highest concentrations were arginine and lysine, and at the lowest concentrations were histidine and isoleucine, other studies (65, 67) reported leucine, valine, and arginine at the highest concentrations, and histidine, phenylalanine, and methionine at the lowest concentrations. Similarly, the amino acid content of shrimp hydrolysate is within the wide range of values previously reported [345 g kg^−1^ DM (68) to 862 g kg^−1^ DM (62)], but its amino acid profile varies from comparable sources earlier reported. Indeed, whereas, in the current study, the essential amino acids found at the highest concentrations were arginine and lysine, and at the lowest concentrations were histidine and methionine, in other studies (62, 68) arginine, isoleucine, leucine and lysine were present at the highest concentrations and at the lowest concentrations were histidine, phenylalanine, and methionine. The variations observed between studies on CP and AA contents might be explained by the well-known effects of species, growth stage, feeding conditions, part of the animal used (e.g., head, cephalothorax, in shrimp, or head, tentacles, viscera, in squid), conditions of processing and hydrolysis, including enzymes applied, duration, temperature, and pH, and storage, among others (61, 64, 65).

According to the National Research Council (63), methionine is frequently the most limiting amino acid in protein sources routinely used in petfood, such as poultry by-product meal, meat and bone meal, and soybean meal prompting manufacturers to employ synthetic methionine supplements (69). The high methionine content of shrimp hydrolysate and, especially, of squid meal suggests the potential of these by-products to overcome the dietary deficiency in this essential amino acid.

### 4.2 Antioxidant activity

The evaluation of the antioxidant activity of novel protein sources contributes to the understanding of their functional potential for animal health. As the response of antioxidants to different radicals or oxidants may vary, leading to a lack of consensus on the optimal method for demonstrating antioxidant activity (70, 71), four distinct antioxidant assays were used in the present study: ABTS, DPPH, FC and FRAP. ABTS assay relies on a hydrogen atom transfer (HAT) or a single-electron transfer (SET) mechanism and it quantifies the amount of ABTS• + radical cation quenched and the residual ABTS + concentration (72), and can be performed in a wide pH range and in hydrophilic and lipophilic samples (73). DPPH assay relies on a SET mechanism, whereas the DPPH• is scavenged, which is affected by the solvent and the pH of the reaction (74, 75). Folin–Ciocalteu assay is also based on a SET mechanism, measuring the reducing capacity of compounds in an alkaline medium, but only applicable to hydrophilic solvents (73, 76). Lastly, FRAP assay evaluates the capacity of compounds to reduce the ferric 2,4,6-tripyridyl-s-triazine complex through a SET mechanism in an acidic medium (77). Shrimp hydrolysate extracts showed higher antioxidant activity in all methods tested in comparison to squid meal extracts. The reaction of squid meal extracts in the DPPH assay was below the threshold of detection, requiring higher concentrations for the reaction to occur (data not shown), as previously reported (73). Although bioactive compounds are known to exert antioxidant properties, scavenging free radicals, thus preventing their harmful effects on health (78, 79), additional research is needed to identify the specific compounds responsible for the observed antioxidant activity in these extracts.

The antioxidant activity of squid meal extracts was assessed for the first time, thus precluding the comparison of the results herein obtained with the literature. Earlier research has demonstrated the antioxidant properties of components from *D. gigas* with higher antioxidant activity of arms collagen hydrolysates in contrast to fins collagen hydrolysates (80), and higher antioxidant activity of skin gelatine in contrast to gelatine from arms and fins (81). The antioxidant activity of *L. vannamei* shrimp hydrolysates has been demonstrated before (82, 83), but the variety of the process of hydrolysis, along with the diversity of methodology, such as the extraction and the method of antioxidant activity used, makes it difficult to compare findings among studies. Hydrolyzed peptide size may contribute to the antioxidant properties of shrimp hydrolysate (84, 85), with smaller fractions exhibiting the highest activity (85, 86). Moreover, other compounds present in shrimp, such as phenolic compounds, might contribute to the antioxidant activity (87, 88). Additionally, as food processing influences the antioxidant properties (89), more research is needed to evaluate the impact of dog food extrusion on the antioxidant potential of novel protein sources.

### 4.3 Palatability

The palatability of novel ingredients is of crucial importance as it can affect food consumption. Thus, the addition of palatability enhancers is a common practice in the petfood industry, being protein hydrolysates extensively employed for this purpose (90). Palatability refers to taste, odor, and mouth feel (texture, shape, and size) (91), and it is influenced by the composition of diets, such as the content of DM, fiber, carbohydrates, fat and protein (92). In the present study, although no food refusal was observed, the palatability of diets was negatively affected by the inclusion of 150 g kg^−1^ of shrimp hydrolysate or squid meal in place of the basal diet. This result was unexpected as both squid meal and shrimp hydrolysate presented high levels of glutamic and aspartic acids, two amino acids found in various foods, including seafood, known to induce umami, a taste highly attractive to dogs (93, 94). Indeed, earlier studies found no negative or even positive effects on palatability with higher inclusion levels of marine by-products than the level used in the current study (12, 95). The lower palatability of diets with 15% inclusion of squid meal or shrimp hydrolysate might be due to protein sources being mixed with the basal diet immediately before being offered to dogs, instead of included in the kibble, as previously shown with microalgae supplementation (11). Future research should be performed to evaluate the palatability of diets containing squid meal and shrimp hydrolysate included in the extruded complete diet.

### 4.4 Body weight, food intake, fecal output and characteristics, *in vivo* digestibility, and metabolizable energy

To the best of authors knowledge this is the first study that assessed the *in vivo* effects of inclusion of squid meal and shrimp hydrolysate in diets for dogs, so the results obtained here cannot be compared with the literature. Based on studies performed with other monogastric species (96, 97), and taken into consideration the results obtained in the palatability trials, the levels of dietary inclusion in the digestibility trials were set at 50, 100, and 150 g kg^−1^. As daily food allowance was defined according to the ME requirements and adjusted to the ideal BW, inclusion levels of squid meal and shrimp hydrolysate did not affect BW. Diet intake was not affected by the level of squid meal, but increasing levels of shrimp hydrolysate linearly increased diet intake, while comparing to the basal diet, both studied protein sources increased food intake. However, these effects lack biological meaning. Differences in intake of nutrients and energy reflect the different chemical composition of the basal and experimental diets in both experiments.

The number of defecations, fecal DM content and consistency score, parameters highly valuable for dog owners, were not affected by increasing levels of squid meal and shrimp hydrolysate inclusion. Compared to the basal diet, although differences reached significance on consistency score, feces were all classified between soft, shaped, and moist stools leaving spots on the floor (3.0) and approximately firm, shaped, and dry stools (3.5), which is considered the optimum (98). The higher fecal output observed with the basal diet relative to the diets with the novel protein sources, reflects the modest digestibility of this diet.

Along with fecal output and quality, diet digestibility assumes relevant importance to pet owners. In experiment 1, the ATTD of CP was the highest with 150 g kg^−1^ inclusion of squid meal. Despite being known that indigestible protein might reduce fecal quality due to the high osmotic pressure promoted by the fermentation of protein by proteolytic bacteria (99), the effect on CP ATTD was not reflected in fecal DM and consistency score. Although, the decreased molecular weight resulting from hydrolysis of protein sources is expected to improve CP ATTD, the inclusion of increasing levels of shrimp hydrolysate had no effect on this parameter. Compared to the basal diet, the inclusion of the studied protein sources increased ATTD of DM, nutrients, and energy, with the major difference being observed for CP. This demonstrates the high potential of these ingredients to be included in highly digestible diets for dogs. Moreover, despite not being herein studied the allergic responses to squid meal and shrimp hydrolysate, these novel protein sources can also have the potential to be included in therapeutic diets to prevent adverse food reactions (21).

### 4.5 Fecal end-fermentation products

Despite the significant differences observed among squid meal inclusion levels and between basal diet and squid meal supplementation, fecal pH was nearly neutral in both experiments. These high fecal pH values are known to favor the fermentation of undigested protein in the colon (100), generating iso-butyrate, iso-valerate and ammonia-N (101) that might have negative effects on gut health and fecal odor (100). In the current study, despite the observed differences in CP ATTD with increasing levels of squid meal, no effects were observed on these parameters. Compared to the basal diet, the inclusion of squid meal increased ammonia-N and slightly reduced fecal quality, suggesting a higher amount of protein reaching the large intestine. Indeed, the higher CP intake observed with squid meal inclusion in relation to the basal diet could have resulted in higher protein fermentation in the colon, culminating in higher ammonia-N levels (102). Moreover, the inclusion of squid meal decreased fecal butyrate concentration when compared to the basal diet. As this VFA constitutes the main source of energy for colonocytes, thus contributing for the maintenance of cell growth and differentiation in the gut, its reduction might suggest a lower preventing role in inflammation and colon cancer (103). Conversely, a positive correlation between ammonia-N and butyrate concentrations have been previously observed in humans and rats (104, 105). The majority of ammonia-N is produced by bacteria through the deamination of amino acids (101), and can be absorbed by colonocytes or utilized by bacteria for protein synthesis and metabolism (100).

Despite the absence of effects of increasing levels of shrimp hydrolysate on VFA production, when compared to the basal diet, the dietary inclusion of this protein source significantly increased total VFA and individual concentrations except for butyrate, tended to decrease fecal pH, and did not influence ammonia-N concentration. An earlier *in vitro* study has also reported increased production of VFA with hydrolyzed soy protein, such as acetate, iso-butyrate and iso-valerate, compared to non-hydrolyzed soy protein (106). The high concentrations of acetate and propionate observed with shrimp hydrolysate inclusion diets might benefit dogs health, namely by regulating host metabolic, immune and neuro-immunoendocrine responses (107–109), and lowering cholesterol (110), being also observed decreased acetate and propionate concentrations in dogs diagnosed with chronic enteropathy in comparison to healthy dogs (111). On the other hand, the increased iso-butyrate and iso-valerate concentrations, known to be originated from the bacterial fermentation of leucine, isoleucine and valine (112), results in increased concentration of fermentation products, such as ammonia-N, that might be detrimental to host health (100).

### 4.6 Fecal microbiota

The fecal microbiota is a complex ecosystem influencing host health by modulating the immune system (113, 114), and also by regulating nutrient utilization of substances entering the colon, where the production of fecal metabolites occurs (10). While there are some variations of microbiota along the dog intestinal tract (115, 116), fecal microbiota is mostly studied due to its ease of sampling and non-invasiveness (117). Changes in diet composition, such as protein content and source, are normally accompanied by variations in the microbiome profile of the gut (118), within a short period of time, namely 2 days for metabolites, such as VFA and ammonia-N, and 6 days for microbiota (119). In the current study, the inclusion of squid meal or shrimp hydrolysate in the diets did not significantly affect the beta and alpha diversity and relative abundance of bacteria. All diets presented higher abundances of *Turicibacter,* Peptostreptococcaceae, and *Blautia*, all pertaining to the phylum Firmicutes the most common phylum in dog gut (118). Higher levels of *Turicibacter* and *Blautia* are indicators of a healthy gut microbiota, while lower levels of these genera have been observed in dogs diagnosed with chronic inflammatory enteropathy (120).

The inclusion of 150 g kg^−1^ of shrimp hydrolysate increased the abundance of a genus pertaining to Oscillospiraceae. A positive correlation of *Oscillospira*, a genus from Oscillospiraceae, with the production of acetate, butyrate, propionate (121), and valerate (122) has been shown, thus suggesting the potential of shrimp hydrolysate as a prebiotic (121). The inclusion of 100 g kg^−1^ of shrimp hydrolysate decreased the abundance of *Lactobacillus* that is known to have the ability to cross-feed other commensals to produce butyrate (123) and was earlier reported to decrease when dogs are fed diets high in protein and fat obtained from natural sources (124). However, in the current study, the effect of shrimp hydrolysate inclusion on butyrate concentration did not reach significance and diet SH10 presented a lower CP content than diet SH15, thus being not clear the mechanism for a decreased *Lactobacillus* genus with this diet. The inclusion of 50 g kg^−1^ of shrimp hydrolysate decreased the abundance of a genus pertaining to Firmicutes, in agreement with a previous study showing a decrease in Firmicutes abundance when dogs were fed a protein hydrolysate from leather waste (125). Conversely, the increased concentration of acetate, butyrate and propionate have been associated with the increased relative abundance of Firmicutes (126). Further research is needed to fully understand the effects of shrimp hydrolysate on microbiota profile.

## 5 Conclusion

The present study demonstrated, for the first time, the potential of squid meal and shrimp hydrolysate as novel protein sources for dog nutrition. Both by-products presented higher protein and methionine contents than commonly used protein sources, and their dietary inclusion increased diet digestibility, suggesting their potential to be included in high protein digestible diets. Despite the general absence of effects of inclusion levels on fecal metabolites, and conversely to squid meal, feeding dogs with shrimp hydrolysate diets affected microbiota composition. In summary, the findings support the potential of these protein rich by-products for dog feeding. However, additional research is needed to fully evaluate their functional properties.

## Data availability statement

The datasets presented in this study can be found in online repositories. The names of the repository/repositories and accession number(s) can be found at: https://www.ebi.ac.uk/ena, PRJEB71521.

## Ethics statement

The animal study was approved by Animal Ethics Committee of School of Medicine and Biomedical Sciences, University of Porto. The study was conducted in accordance with the local legislation and institutional requirements.

## Author contributions

JG-F: Formal analysis, Investigation, Writing – original draft. TA: Resources, Writing – review & editing. AF: Funding acquisition, Writing – review & editing. TY: Investigation, Writing – review & editing. AC-S: Investigation, Writing – review & editing. SL: Supervision, Writing – review & editing. MM: Conceptualization, Formal analysis, Investigation, Supervision, Writing – review & editing. AC: Conceptualization, Formal analysis, Funding acquisition, Investigation, Project administration, Resources, Supervision, Writing – review & editing.

## Footnotes

^1^https://github.com/najoshi/sabre
